# Oxidative Stress in Chronic and Age-Related Diseases

**DOI:** 10.3390/antiox11030521

**Published:** 2022-03-08

**Authors:** Chiara Mozzini, Mauro Pagani

**Affiliations:** Department of Medicine, ASST Mantova, C. Poma Hospital Strada Lago Paiolo, 10, 46100 Mantova, Italy; mauro.pagani@asst-mantova.it

The connection between oxidative stress and common age-related diseases presents an exciting field of research. Oxidative stress is involved in several age-related conditions (cardiovascular diseases, chronic obstructive pulmonary disease, chronic kidney disease, neurodegenerative diseases, and cancer, as well as sarcopenia and frailty). This Special Issue discusses preclinical and clinical evidence highlighting the central role of oxidative stress in the progression of specific diseases.

Culmsee et. al’s study [1], which reported that Cimicifuga racemosa extract Ze 450 promoted sustained cellular resilience to oxidative stress in vitro and in vivo, was particularly intriguing. In previous research, they demonstrated that Ze 450 had additional beneficial effects on age-related and metabolic diseases. In this study, they focused on the treatment of menopausal conditions, investigating the molecular mechanisms underlying the effects of Ze 450 on ferroptosis in neuronal cells, with a particular focus on mitochondria. Culmsee and colleagues found that Ze 450 directly inhibited complex I activity in mitochondria and enhanced the metabolic shift towards glycolysis. A well-established in vitro model system of oxidative stress was used to mimic the imbalance of redox homeostasis during the menopausal transition. Ze 450 and metformin were compared in vivo to assess their impact on longevity under basal conditions and oxidative stress exerted by the mitochondrial toxin paraquat.

Another important topic in this area of research is ischemia–reperfusion (IR) injury, which occurs when blood provision to an organ is diminished or interrupted and then re-established. IR injury is a phenomenon resulting from the reoxygenation of tissues previously subjected to anoxia. Blood flow restoration triggers a sequence of biochemical processes, such as the formation of reactive oxygen species (ROS), the activation of cytokines, and the alteration of capillary permeability; this process exacerbates the cellular dysfunction suffered during the ischemic process, being the most affected tissue in the extremities. Cearra and colleagues [2] evaluated the effectiveness of folinic acid in a rodent model of lower-limb ischemia–reperfusion, emulating the characteristics, the techniques, and the duration of ischemia periods used in clinical practice when performing limb surgery under ischemic conditions. The authors concluded that the administration of folinic acid before tourniquet release could be considered a prophylactic treatment to prevent IR-induced damage. This is a significant finding, mainly because folinic acid is a well-known, inexpensive, and little-described toxicity drug.

Wang and colleagues also contriubutes to this Special Issue [3], finding that acute stroke is accompanied by the production of ROS, inflammation, and transient hyperglycemia. All of these contribute to neuronal injury and are correlated with outcomes in patients with stroke. Their study centered on the IL-6-signaling and Jak2/Stat3 pathway signaling and Jak2, considering its important role in the regulation of immune responses and many pathological processes. The authors used a Jak2 inhibitor, AG490, to explore its effects on inflammation in the cerebral cortical, hepatic, and skeletal muscle tissues and the changes in insulin signaling in a rat model of acute cerebral ischemia. They concluded that IL-6 is a plausible mechanistic link between chronic inflammation and glucose intolerance/insulin resistance via the IL6/Jak2/Stat3 axis. They provided experimental evidence of the suppressive effects of the Jak2 inhibitor AG490 on brain injury, apoptosis, oxidative stress, neuroinflammation, hyperglycemia, glucose intolerance, skeletal/hepatic oxidative stress, skeletal/hepatic inflammation, and insulin resistance. We would also like to highlight the work of Wang and colleagues, who further strengthened the theory that agents or strategies targeting inflammation, hyperglycemia, or both may have promise in preventing disease progression in conditions such as cerebral ischemia.

The study by Döppler and colleagues [4] concerned oxidative stress and inflammation in pancreatic acinar-to-ductal metaplasia (ADM), a reversible process that occurs after pancreatic injury but becomes permanent and leads to pancreatic lesions in the presence of oncogenic mutations. In this study, the authors identified the generation of mitochondrial ROS as a common signaling pathway for the inducers of ADM. Protein kinase D1 (PKD1), a kinase that can be activated by hydrogen peroxide, affects acinar cell survival and mediates ADM, which is in part due to the PKD1 target, the proinflammatory factor nuclear factor kappa-B (NF-κB). Overall, these data implicate ROS-PKD1 signaling as a common feature of different inducers of pancreatic ADM. Since PKD1 links to most transcription factors crucial for the acinar cell dedifferentiation process, this kinase may be a critical and targetable component in the development of strategies to prevent pancreatic cancer.

Cardiovascular pathology is another topic that was necessary to address in this Special Issue. Szabó and colleagues’ study [5] aimed to investigate the role of the endocannabinoid system in the modulation of inflammation and oxidative stress in older female and male hearts, focusing on the CB1 receptor blockade and rimonabant, a selective CB1 receptor antagonist. Beyond the well-known antiobesity effects of rimonabant, its ability to promote anti-inflammatory and antioxidant alterations in different tissues has received significant attention. Understanding the mechanisms that lead to “inflammaging” and oxidative stress is crucial to reducing age-related morbidity. CB1-receptor-blocker rimonabant has a significant role in the mediation of cardioprotective effects in older female and male rats. Two weeks of rimonabant administration resulted in a significant decrease in the levels of inflammatory NF-κB, TNF-α, and MPO and more effectively protected the heart from oxidative damages. The antioxidant HO and glutathione defense systems are important ROS scavengers that were enhanced by rimonabant. Although rimonabant has been withdrawn from the market, it is still an important compound as it targets several mechanisms to protect the heart from age-related damages.

To conclude, we would like to thank all the authors that have contributed to this Special Issue, “Oxidative Stress in chronic and age-related diseases”. We have covered several aspects through the “bench to bedside” approach. We hope that this issue will spark further interest in the field of oxidative stress research on chronic and age-related diseases.
